# Two Consecutive Days of Extreme Conditioning Program Training Affects Pro and Anti-inflammatory Cytokines and Osteoprotegerin without Impairments in Muscle Power

**DOI:** 10.3389/fphys.2016.00260

**Published:** 2016-06-28

**Authors:** Ramires A. Tibana, Leonardo M. de Almeida, Nuno M. Frade de Sousa, Dahan da Cunha Nascimento, Ivo V. de Sousa Neto, Jeeser A. de Almeida, Vinicius C. de Souza, Maria de Fátima T. P. L. Lopes, Otávio de Tolêdo Nobrega, Denis C. L. Vieira, James W. Navalta, Jonato Prestes

**Affiliations:** ^1^Graduation Program on Physical Education, Catholic University of Brasilia Brasilia, Brazil; ^2^Laboratory of Exercise Physiology, Faculty Estacio of Vitoria Vitoria, Brazil; ^3^Medical Sciences, Faculty of Medicine, University of Brasilia Brasilia, Brazil; ^4^UDF-Centro Universitário Brasília, Brazil; ^5^Department of Kinesiology and Nutrition Sciences, University of Nevada Las Vegas, NV, USA

**Keywords:** inflammatory response, weight training, extreme condition, muscle power

## Abstract

The aim of this study was to investigate the effects of two consecutive extreme conditioning program training sessions (24 h apart) designed to enhance work-capacity that involved both cardiovascular and muscular exercises on cytokines, muscle power, blood lactate and glucose. Nine male members of the extreme conditioning community (age 26.7 ± 6.6 years; body mass 78.8 ± 13.2 kg; body fat 13.5 ± 6.2%; training experience 2.5 ± 1.2 years) completed two experimental protocols (24 h apart): (1) strength and power exercises, (2) gymnastic movements, and (3) metabolic conditioning as follows: 10 min of as many rounds as possible (AMRAP) of 30 double-unders and 15 power snatches (34 kg). The same sequence as repeated on session 2 with the following metabolic conditioning: 12 min AMRAP of: row 250 m and 25 target burpees. Serum interleukin-6 (IL-6), IL-10, and osteoprotegerin were measured before, immediately post and 24 h after workout of the day (WOD) 1, immediately post, 24 and 48 h after WOD 2. Peak and mean power were obtained for each repetition (back squat with 50% of 1 repetition maximum) using a linear position transducer measured before, immediately post and 24 h after WOD 1, immediately post and 24 h after WOD 2. Blood lactate and glucose were measured pre and immediately post WOD 1 and 2. Although both sessions of exercise elicited an significant increase in blood lactate (1.20 ± 0.41 to 11.84 ± 1.34 vs. 0.94 ± 0.34 to 9.05 ± 2.56 mmol/l) and glucose concentration (81.59 ± 10.27 to 114.99 ± 12.52 vs. 69.47 ± 6.97 to 89.95 ± 19.26 mg/dL), WOD 1 induced a significantly greater increase than WOD 2 (*p* ≤ 0.05). The training sessions elicited significant changes (*p* ≤ 0.05) in IL-6, IL-10 and osteoprotegerin concentration over time. IL-6 displayed an increase immediately after training WOD 1 [197 ± 109%] (*p* = 0.009) and 2 [99 ± 58%] (*p* = 0.045). IL-10 displayed an increase immediately after only WOD 1 [44 ± 52%] (*p* = 0.046), and decreased 24 and 48 h following WOD 2 (~40%; *p* = 0.018) as compared to pre-exercise values. Osteoprotegerin displayed a decrease 48 h following WOD 2 (~25%; *p* = 0.018) as compared with pre intervention. In conclusion, two consecutive extreme conditioning training sessions increase pro/anti-inflammatory cytokines with no interference on muscle performance in the recovery period.

## Introduction

Extreme conditioning training programs are a growing exercise regimen characterized by high intensity, constantly varied, functional movement (Tibana et al., 2015). Typical workouts include Olympic lifting (snatch, clean, and jerk), power lifting (squat, deadlift, press/push press, bench press), and gymnastic movements (pull-ups, toes-to-bar, knees-to-elbows, lunges, muscle-ups, burpees, dips, push-ups, rope climbs; Tibana et al., 2015). These exercises are often combined into high-intensity workouts that are performed in rapid, successive repetition, with limited or no recovery time.

However, high-intensity training modalities have also been the subject of scrutiny, with concerns of elevated risk of nonfunctional overreaching. Recently, investigators have demonstrated that a CrossFit bout elicited an acute blood oxidative stress response comparable to a traditional bout of high-intensity treadmill running in males with 3 or more months of CrossFit experience (Kliszczewicz et al., 2015). Similarly, Szivak et al. (2013) and Heavens et al. (2014) showed in “sister articles” that a high-intensity short rest protocol (which consisted of a descending pyramid scheme of back squat, bench press, and deadlift, beginning with 10 repetitions of each, then 9, then 8, and so on until 1 repetition on the final set) elicits significant increases in indirect blood markers of muscle damage [myoglobin, inteleukin-6 (IL-6) and creatine kinase] and resulted in exacerbated metabolic (lactate) and adrenal function (cortisol) activation in men and women with experience in resistance training. This prolonged response can lead to compounding physiological stress over consecutive exercise sessions, and can contribute to a nonfunctional overreaching.

Moreover, it has been shown that repeated intense interval exercise (three consecutive days of an intermittent run protocol to exhaustion) elicited significant CD4+, CD8+, and CD19+ lymphocyte cell death and migration after the third day of running. Considering this immune response, it would be recommended to incorporate a rest day following two consecutive days of high-intensity intermittent running to minimize immune cell modulations and reduce potential susceptibility to infections (Navalta et al., 2014). Nonetheless, Tuan et al. (2008) submitted trained runners to treadmill exercise at 85%VO_2_max for 30 min daily over 3 consecutive days and found that high-intensity exercise induced a significant dysfunction of the mitochondrial energy status in peripheral blood immune cells, which was accompanied by an increased propensity for apoptosis and an increase in tumor necrosis factor-alpha.

These alarming physiological responses, training complexity and huge numbers of individuals taking part in such exercise activities increases the need for studies to evaluate the effect of this mode of activity on the immune and metabolic system. Therefore, the aim of the present study was to analyze two consecutive extreme conditioning sessions on IL-6, IL-10, osteoprotegerin, muscle power, blood lactate and glucose in trained men. The initial hypothesis is that two such consecutive sessions exacerbate cytokine responses accompanied by muscle power impairment.

## Methods

### Experimental approach to the problem

**Figure 1:**
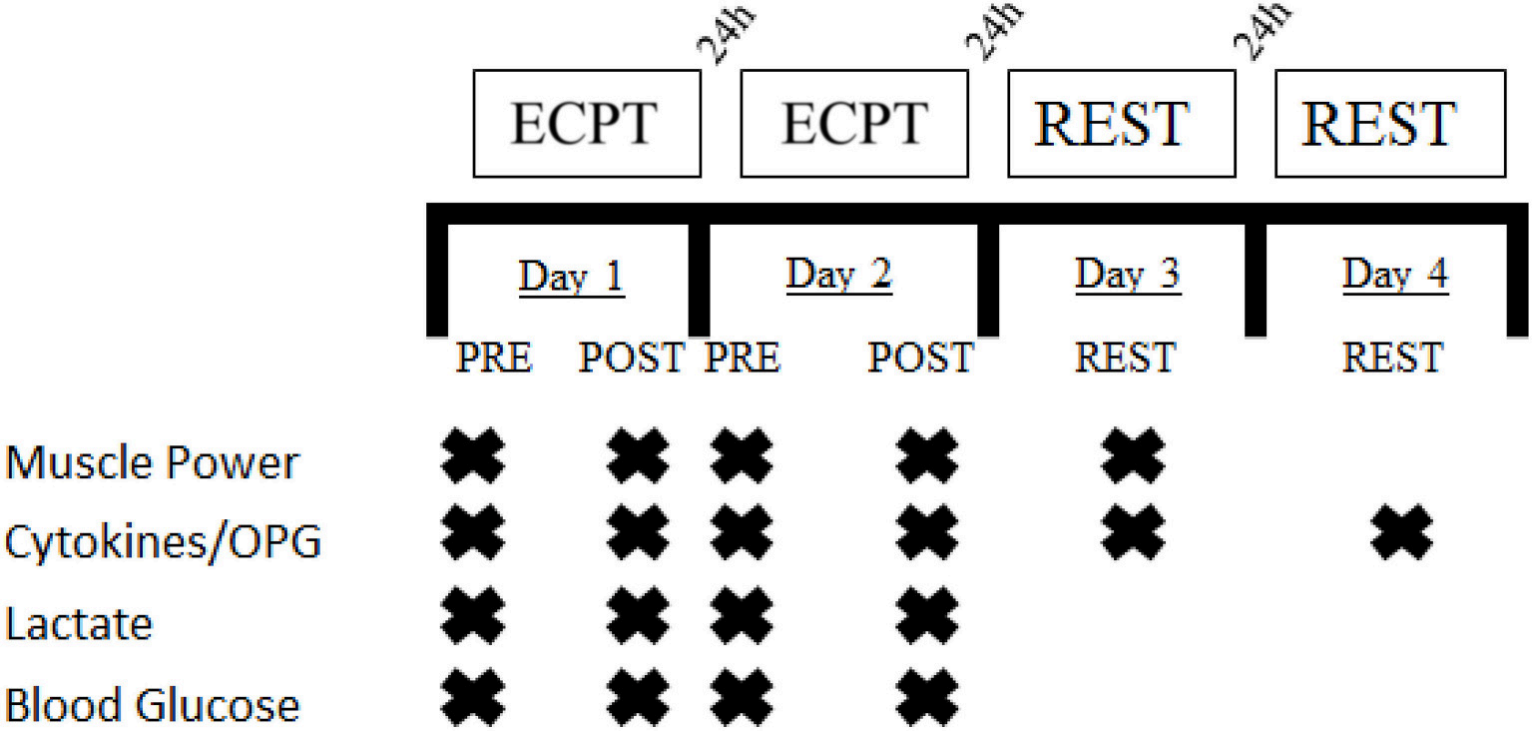
**Experimental design**. ECPT: Extreme Conditioning Program Training.

This study was designed to compare the effects of two different extreme conditioning sessions on cytokines, metabolic and neuromuscular responses in trained, adult men. In this study, the workouts of the day (1 vs. 2) were independent variables, while the dependent variables consisted of the changes in IL-6, IL-10, IL-10/IL-6 ratio, OPG, lactate, blood glucose, and mean power (Figure 1). The responses of cytokines, lactate, blood glucose, and mean power was assessed before and immediately post following workouts of the day and following 24 h (cytokines and mean power) and 48 h (cytokines) after workout of the day 2. The extreme conditioning sessions 1 (predominantly Olympic lifting) and 2 (body weight exercises) consisted of performing strength and gymnastic exercises paired with endurance intensive efforts (Chamari and Padulo, 2015). This design allowed us to individually assess the influence of different extreme conditioning sessions on immune response in our cohort of adult men.

### Subjects

Nine members of the extreme conditioning program community (age 26.7 ± 6.6 years) were recruited through advertisements. Subject characteristics are presented in Table 1. All subjects were free of injury and known illness, were not using drugs to enhance performance, and had a minimum of 6 months of extreme conditioning training. Moreover, they were interviewed by the researcher and reported to have previous experience in resistance strength training and cardiovascular training experience before practicing extreme conditioning workouts. Subjects were advised to refrain from ingesting caffeine and alcohol for 24 h before all tests, avoid any exercise in the 48 h before the experimental sessions, and to maintain their normal daily diet during the study.

**Table 1:** **Subject's physical characteristics**.

	***n* = 9**
Age, year	26.8 ± 6.6
Weight, kg	78.8 ± 13.2
Body fat, %	13.5 ± 6.3
VO2max, mL.(kg. min)−1	49.4 ± 3.3
Back squat 1RM, kg	146.9 ± 23.7
Systolic blood pressure, mmHg	128.6 ± 11.4
Diastolic blood pressure, mmHg	74.4 ± 6.9
Resting heart rate, bpm	73.4 ± 11.9

*VO_2_max, maximal oxygen uptake*.

All participants signed an informed consent document and the study was approved by the University Research Ethics Committee for Human Use and conformed to the Helsinki Declaration on the use of human subjects for research.

### Training session days

Subjects completed 2 training sessions 24 h apart (Table 2). In workout of the day 1, subjects completed: (a) five sets of one repetition of snatch from the block at 80% of one-repetition maximum (1RM) with 2–5 min of rest intervals; (b) 3 sets of 5 Touch & Go Snatches (full) at 75% of 5RM with 90 s of rest between sets; (C) 3 sets of 60 s of weighted plank hold with 90 s of rest; After the third set of the aforementioned exercises, 5 min of rest was allowed and then endurance conditioning was performed with 10 min of as many rounds as possible (AMRAP) 30 double-unders and 15 power snatches (34 kg; Padulo et al., 2015).

**Table 2:** **Schematic representation of the training sessions**.

	**Strength**	**Gymnastic**	**Metabolic conditioning**
WOD 1	(1) 5 × 1 Snatch from blocks (just above knee) 80% of 1RM from blocks—2–5 min of rest. (2) 3 × 5 Touch & Go Snatches (full) @ 75% of 5RM—90 s of rest.	3 × 60 s Weighted Plank Hold (plate on back)—heaviest possible, 90 s of rest.	10 min AMRAP of: 30 Double-Unders 15 Power Snatches
WOD 2	(1) 5 × 1 Clean from blocks (just above knee) 80% 1RM—2 min of rest. (2) 5 × 1 Jerk from blocks / 80% of 1RM from blocks—2 min of rest. (3) (1a) 3 × 5 Touch & Go Cleans (full) / 70% of 5RM / 90 s of rest.	(1b) 3 × 10 Strict Hand Standing Push up (as fast as possible)—2 min of rest.	12:00 AMRAP of: Row 250 m 25 6″ Target Burpees

*WOD, Workout of the day; AMRAP, as many round as possible*.

In workout of the day 2, subjects completed: (a) five sets of one repetition of clean and Jerk from the block at 80% of 1RM with 2–5 min rest intervals; (b) 3 sets of 5 touch and go cleans (full) with 70% of 5RM with 2–5 min rest; (c) 3 sets of 10 strict hand standing push-ups; After the third set of the aforementioned exercises, 5 min of rest was allowed, followed by endurance conditioning with 12 min AMRAP of rowing (250 m, Concept) and 25 target burpees.

The goal of the endurance conditioning sessions were to complete each training session in the quickest time possible, without compromising exercise technique.

### Cytokines and blood lactate and glucose concentration

Participants reported to the laboratory between 08:00 and 10:00 a.m., and blood samples (15 mL) were drawn from the antecubital vein into vacutainer tubes (Becton Dickinson, Brazil). Samples were then centrifuged at room temperature at 2500 rev. min^−1^ for 15 min. All subjects were encouraged to avoid smoking, alcohol and caffeine consumption to avoid influence on these parameters. The serum was removed and frozen at −80°C for further analysis. Serum was analyzed for amyloid A using a DADE Dimension RXL clinical chemistry analyzer (Dade-Behring, Inc, Newark, DE, USA). The analyzer was calibrated daily using Liquid-Assayed Multiqual (Bio-Rad, Hercules, CA, USA), and two levels of quality control with known concentrations. In addition, serum IL-10 and IL-6 were assessed using commercially available enzyme-linked immunosorbent assay (ELISA) kits (BioLegend's ELISA Max Deluxe, San Diego, CA, USA) and osteoprotegerin (R&D System Inc., Minneapolis, MN, USA). Standard curves were generated using commercially available microplate reader-compatible statistical software (MicroWin 2000, Microtek Laborsysteme GmbH, Overath, Germany). All samples were determined in duplicate to guarantee the precision of the results. For all measures the mean intra-assay coefficient of variation was 2.9–9.5%, the inter-assay coefficient of variation was 5.9–7.0%, and the sensitivity was 0.0093 pg/mL.

Standard procedures were followed for blood lactate and glucose collection, management and analysis (Goodwin et al., 2007). Blood samples (25 μL) were collected from the earlobe during a rest period (before) and after each experimental procedure. Blood lactate and glucose concentration was determined by electroenzymatic method (1500 Sport; Yellow Springs Instruments Inc., Yellow Springs, OH, USA).

### Muscular power output

The power produced during each experimental session was measured by a linear position transducer (Peak Power, Cefise, Sao Paulo, Brazil). The configurations for test assessment and calibration followed the manufacturer's specifications. The equipment was attached to the barbell during the back squat (5 repetitions of 50% of 1RM; Cormie et al., 2007; Tibana et al., 2016) to register the time and the displacement at a frequency of 100 Hz. Subsequently, peak and average power (watts) produced during the concentric contraction were determined by the manufacturer's software (version 4.0.4.6; Peak Power software analysis).

### Statistical analysis

The data are expressed as mean value and standard deviation (SD). The Shapiro-Wilk test was applied to check for normality distribution of study variables. A repeated measures ANOVA was used to compare cytokines, OPG and power between time points. Compound sphericity was verified by the Mauchley test. When the assumption of sphericity was not met, the significance of *F*-ratios was adjusted according to the Greenhouse-Geisser procedure. Tukey's *post-hoc* test with Bonferroni adjustment was applied in the event of significance. Paired sample *t*-tests were used to compare cytokines and OPG changes (post–pre and % of change) between training sessions. Pearson's correlation was used to explore the relations between cytokines and power variables. Based on alpha error of 0.05 and a power (1 – β) of 0.80, the sample effect size *f* was 0.65 for IL-6, 0.32 for IL-10, 0.49 for IL-10/IL-6 ratio, and 0.20 for OPG. The level of significance was *p* ≤ 0.05 and SPSS version 20.0 (Somers, NY, USA) software was used.

## Results

**Figure 2:**
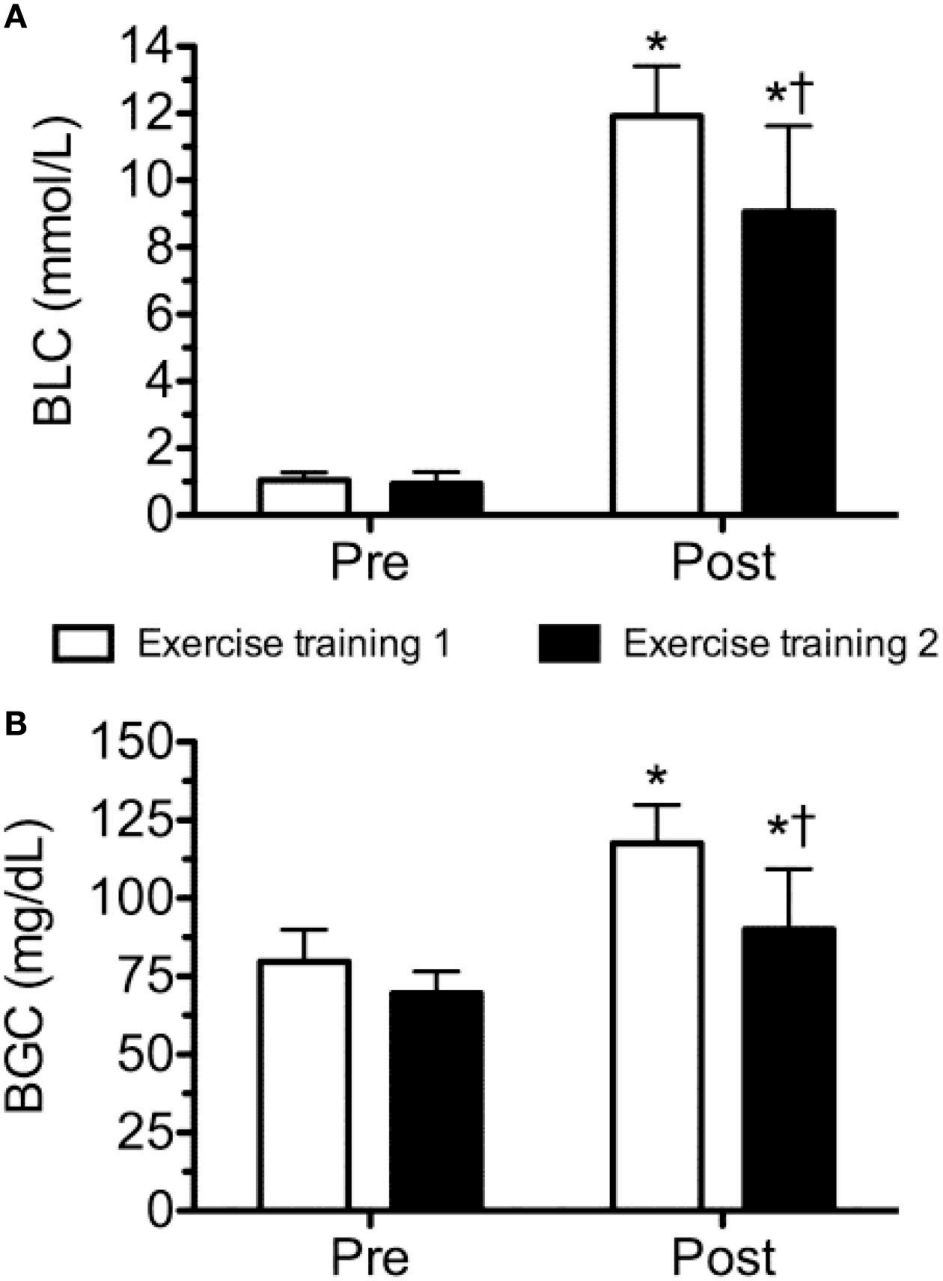
**Blood lactate (BLC; A) and glucose (BGC; B) concentration before and after workout of the day 1 and workout of the day 2**. Values are expressed as means ± *SD*. ^*^p ≤ 0.05 to Pre; ^†^*p* ≤ 0.05 to Post training session 1.

The physical characteristics of the subjects are presented in Table 1. Body fat and blood pressure were considered to be within normal range. Although both sessions of exercise elicited a significant increase in blood lactate and glucose concentrations, exercise training 1 induced a significantly higher increase than exercise training 2 (Figure 2).

**Figure 3:**
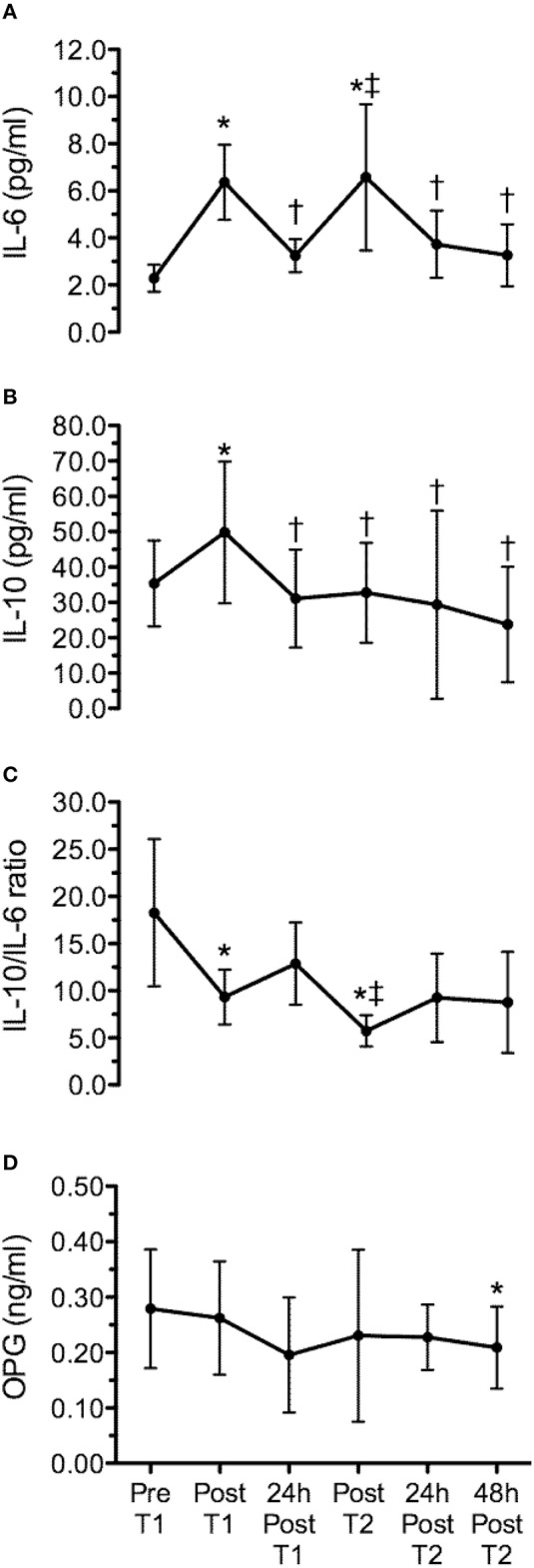
**Time line of interleukin-6 (IL-6) (A), interleukin-10 (IL-10) (B), IL-10/IL-6 ratio (C) and osteoprotegerin (OPG) (D), corresponding to pre (Pre T1) and post workout of the day 1 (Post T1), 24 h after workout of the day 1 (24 h post T1), post workout of the day 2 (Post T2), 24 h (24 h post T2) and 48 h (48 h post T2) after workout of the day 2**. Values are expressed as means ± SD. ^*^p < 0.05 comparing to Pre T1; ^†^*p* < 0.05 comparing to Post T1; ^‡^*p* < 0.05 comparing to 24 h Post T1.

Figure 3 presents the time line of cytokines and OPG, corresponding to pre and post WOD 1, 24 h after WOD 1, post WOD 2, 24 h and 48 h after WOD 2. The training sessions elicited statistically significant changes (*p* ≤ 0.05) in IL-6, IL-10, IL-10/IL-6 ratio, and OPG concentration over time. IL-6 concentration presented a statistically significant increase immediately after WOD 1 (*p* = 0.009) and 2 (*p* = 0.045). However, 24 h after WOD 1 and 2, IL-6 concentration was not statistically significant different (*P* > 0.05) from pre intervention values (Figure 3A). IL-10 concentration presented a statistically significant increase immediately after only WOD 1 (*p* = 0.046). But 24 h after WOD 1, post WOD 2, 24 h and 48 h after WOD 2, IL-10 concentrations was not statistically significant different (*P* > 0.05) from measures obtained pre intervention (Figure 3B). IL-10/IL-6 ratio had a statistically significant decrease (*p* ≤ 0.05) after WOD 1 and 2. There was a tendency toward (*p* = 0.066) lower values of IL-10/IL-6 ratio immediately after WOD 2 when compared to training session 1. IL-10/IL-6 ratio after 24 and 48 h of WOD 2 were not statistically significant different (*P* > 0.05) from pre intervention (Figure 3C). Lastly, OPG concentration 48 h after WOD 2 was significantly lower (*p* = 0.012) than the pre intervention concentration (Figure 3D). When comparing the training responsiveness, there were no significant differences (*p* > 0.05) on absolute (post–pre training session) or percentage (%) changes between training sessions for cytokines and OPG (Table 3).

**Table 3:** **Change (post–pre) in interleukin-6 (IL-6), interleukin-10 (IL-10), IL-10/IL-6 ratio, and osteoprotegerin (OPG) after workout of the day 1 and workout of the day 2**.

	**Workout of the day 1**	**Workout of the day 2**
IL-6, pg/mL	4.1 ± 1.9 [197 ± 109%]	3.3 ± 2.6 [99 ± 58%]
IL-10, pg/mL	14.4 ± 17.8 [44 ± 52%]	21.4 ± 69.9 [21 ± 70%]
IL-10/IL-6 ratio	−8.9 ± 5.9 [−43 ± 24%]	−7.1 ± 5.8 [−49 ± 27%]
OPG, ng/mL	−0.02 ± 0.09 [−11 ± 45%]	0.04 ± 0.09 [19 ± 39%]

**Figure 4:**
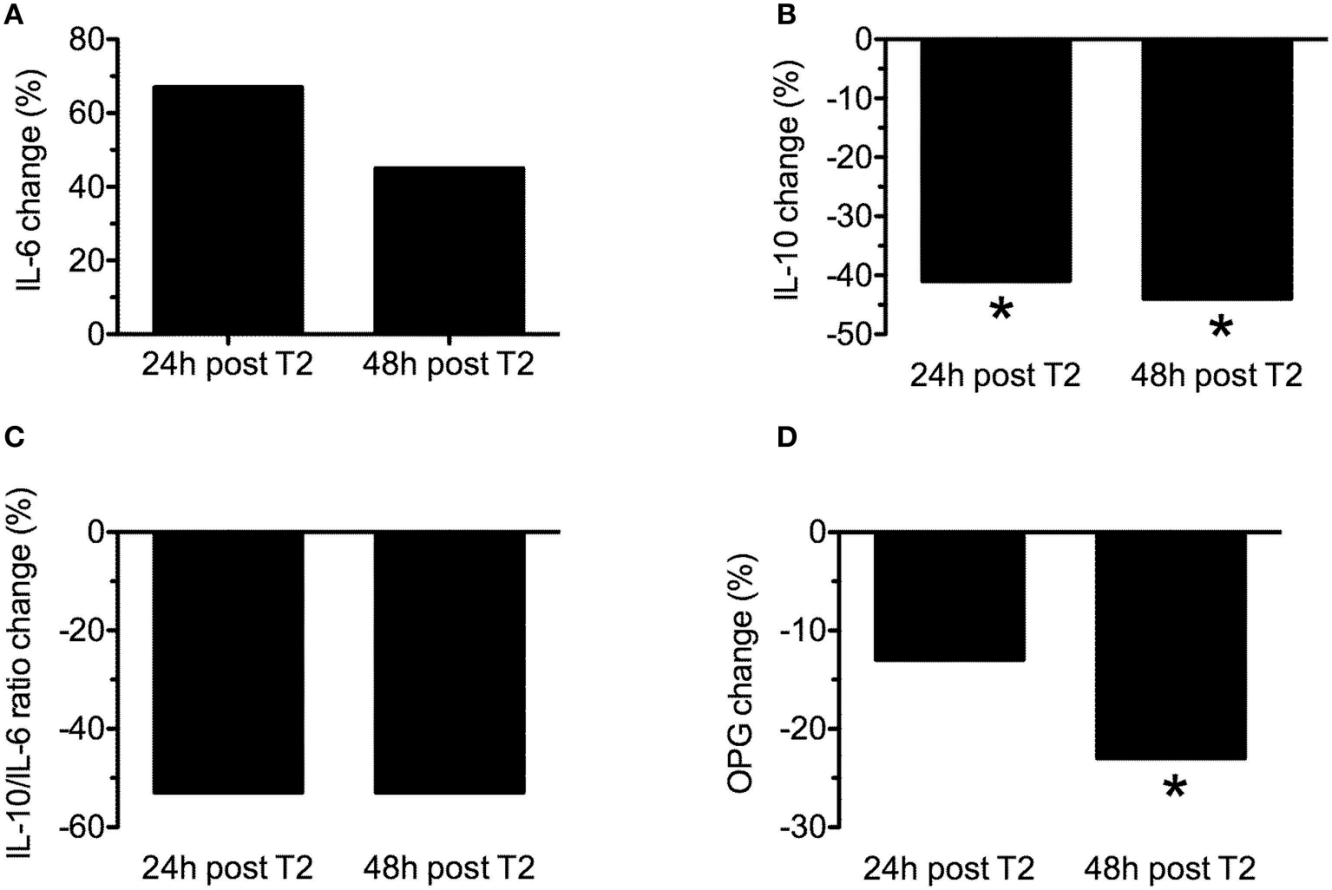
**Mean change (% form baseline) of interleukin-6 (IL-6) (A), interleukin-10 (IL-10) (B), IL-10/IL-6 ratio (C) and osteoprotegerin (OPG) 24 h (24 h post T2) and 48 h (48 h post T2) after workout of the day 2**. ^*^p ≤ 0.05 to baseline.

The mean change (% from baseline) of cytokines and OPG 24 h and 48 h after WOD 2 are shown in Figure 4. IL-10 concentration presented a statistically significant decrease (~40%; *p* = 0.018) 24 and 48 h after WOD 1. OPG concentration had a statistically significant decrease (~25%; *p* = 0.018) only 48 h after WOD 2. The changes of IL-6 and IL-10/IL-6 ratio were not statistically significant. There was a statistically significant correlation between IL-10 and OPG 24 h after WOD 2 (*r* = 0.833; *p* = 0.039).

**Figure 5:**
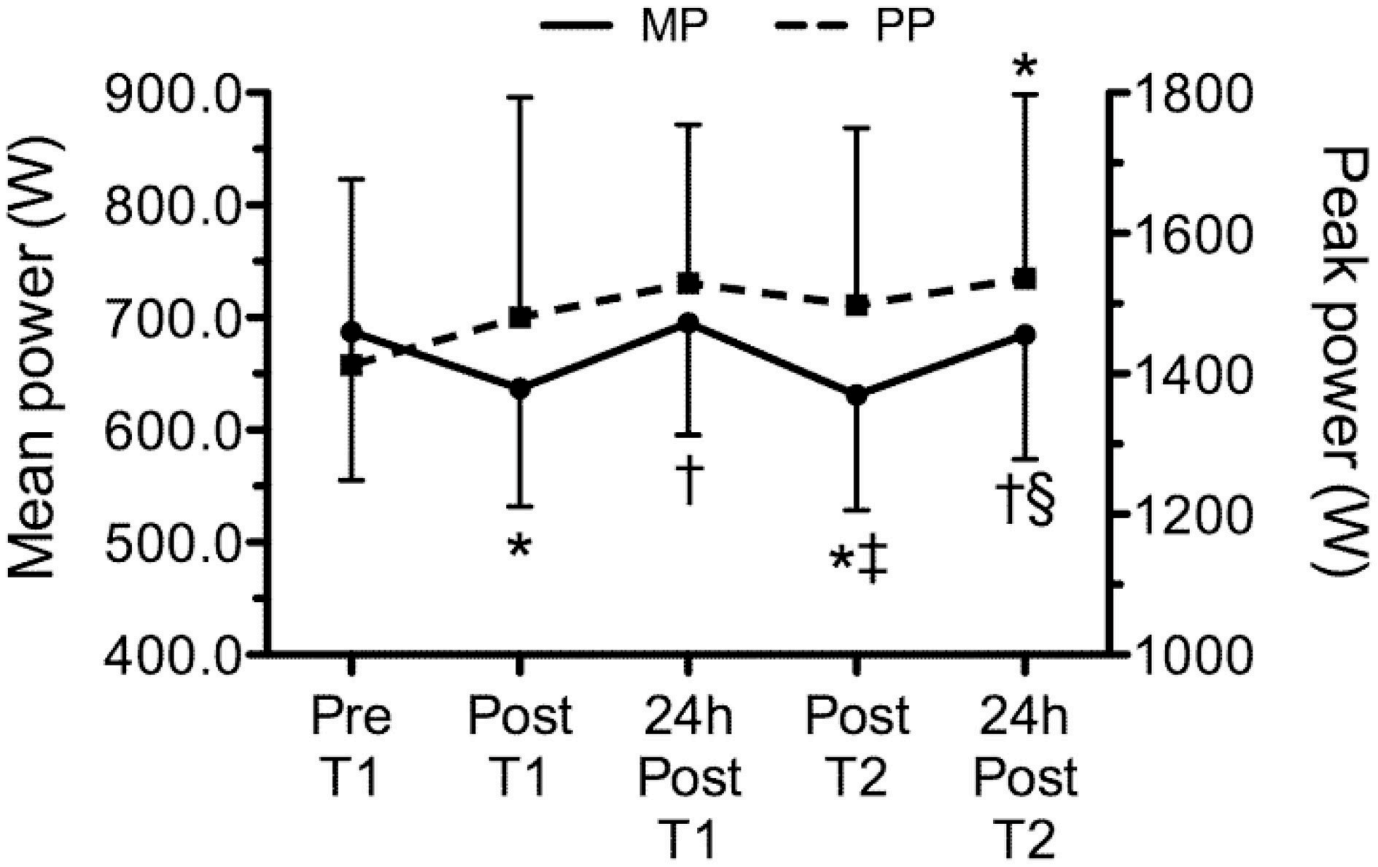
**Time line of mean and peak power, corresponding to pre (Pre T1) and post training session 1 (Post T1), 24 h after workout of the day 1 (24 h post T1), post workout of the day 2 (Post T2) and 24 h after workout of the day 2 (24 h post T2)**. Values are expressed as means ± SD. ^*^p ≤ 0.05 to Pre T1; ^†^*p* ≤ 0.05 to Post T1; ^‡^*p* ≤ 0.05 to 24 h Post T1; §*p* ≤ 0.05 to Post T2.

Figure 5 presents the time line of power, corresponding to pre and post WOD 1, 24 h after WOD 1, post WOD 2 and 24 h after WOD 2. Mean power had a statistically significant decrease (*p* < 0.05) immediately after the training sessions. However, 24 h after, mean power was not statistically significant different (*p* > 0.05) from pre intervention. Peak power was statistically significant higher (*p* < 0.05) 24 h after WOD 2 than pre intervention. No correlations were observed (*p* > 0.05) between cytokines or OPG and power variables.

## Discussion

The main findings of this study were: (a) a single bout of extreme conditioning training provoked high metabolic responses following both sessions, as reflected by significant increases in lactate and glucose concentrations; (b) the training sessions elicited significant increases in IL-6 (WOD 1: 197 ± 109% and WOD 2: 99 ± 58%), IL-10 displayed an increase immediately after WOD 1 (44 ± 52%) and decreased 24 and 48 h following WOD 2, while OPG decreased 48 h after WOD 2; (c) although not statistically significant, IL-10/IL-6 decreased 24 h (~50%) and 48 h (~50%) after WOD 2 when as compared with baseline; (d) the increase in pro/anti-inflammatory cytokines following extreme conditioning training sessions was not accompanied by a decline in muscle power 24 h after WOD 2, partially confirming the initial hypothesis.

To the best of our knowledge, this is the first study to analyze the effects of two different extreme conditioning training sessions on metabolic response (lactate and blood glucose), cytokines (IL-6, IL-10, and Osteoprotegerin) and muscle power in trained men, lending utility to the exercise prescription for preventing nonfunctional overreaching. The results reported in the present study are in agreement with other investigations that observed significant metabolic and inflammatory stress following extreme conditioning training. For example, Szivak et al. (2013) and Heavens et al. (2014) showed in companion articles that a high-intensity with short rest protocol (which consisted of a descending pyramid scheme of back squat, bench press, and deadlift, beginning with 10 repetitions of each, then 9, then 8, and so on until 1 repetition on the final set) elicits a significant increase in muscle damage (myoglobin and creatine kinase), inflammation (IL-6 immediately post of exercise for men: ~3 pg/mL; women: ~3.5 pg/mL) and produced hyperreactions in metabolic (lactate immediately post exercise for men: ~14 Mmol.L-1; women: ~9.1 mmol.L-1) and adrenal function (cortisol) in men and women with experience in resistance training, but not in extreme conditioning training.

Furthermore, Kliszczewicz et al. (2015) found that the CrossFit bout (“Cindy” protocol consisting of as many rounds possible of 5 pullups, 10 push-ups, and 15 air-squats in 20 min) elicited an acute increased on blood oxidative stress (lipid peroxides 1 h post-exercise: CrossFit = ~+143% vs. Treadmill = ~+115%), a decrease on total enzymatic antioxidant capacity immediately post exercise (CrossFit = ~−10% vs. Treadmill = ~−12%), produced high cardiovascular demands at 20 min (CrossFit = ~97% of maximum heart rate vs. Treadmill = ~93% of maximum heart rate) and resulted in a greater rating of perceived exertion (CrossFit = ~9 vs. Treadmill = ~7) response comparable to a traditional bout of high-intensity treadmill running (run at a minimum intensity of the 90% maximal HR) in males with a minimum of 3 months CrossFit training experience.

Similarly, in the present study both extreme conditioning training sessions elicited significant increases in IL-6 (~6 pg/mL) and lactate (1.20 ± 0.41 to 11.84 ± 1.34 and 0.94 ± 0.34 to 9.05 ± 2.56 mmol/l) response comparable with the results of Heavens et al. (2014) (IL-6 immediately post of exercise for men: ~3 pg/mL) and Szivak et al. (2013) (lactate immediately post of exercise for men: ~14 mmol/l). However, different from the present study, Szivak et al. (2013) and Heavens et al. (2014) used subjects with no experience with extreme conditioning training and the protocol was limited to a single training session, which does not accurately reflect the general daily practice of such training. In response to exercise, circulating levels of IL-6 increase up to 100-fold, when long duration and high intensity exercise is performed (Ostrowski et al., 2000; Suzuki et al., 2003; Reihmane et al., 2013). Initially, IL-6 was considered as an immunomodulatory cytokine produced predominantly by leukocytes as an inflammatory response to exercise-induced muscle damage (Bruunsgaard et al., 1997). When it was shown that IL-6 release can be affected by the bioavailability of carbohydrates (Nieman et al., 2003), it was suggested that IL-6 could improve skeletal muscle energy supply (Petersen and Pedersen, 2005). Moreover, exercise provokes an increase primarily in IL-6, followed by an increase in IL-1ra and IL-10 (Petersen and Pedersen, 2005).

Interleukin-10 (IL-10) is an anti-inflammatory cytokine normally released locally from immune cells to help resolve inflammation, and is best characterized for its ability to inhibit macrophage activation (Moore et al., 2001). IL-10 also prevents cytokine synthesis by posttranscriptional mechanisms, as shown in human macrophages where the inhibition of IL-1α, IL-1β, and TNF-α release induced by LPS is a direct consequence of mRNA degradation of their corresponding genes (Petersen and Pedersen, 2005). In this sense, in the present study the response of IL-10 concentration presented a statistically significant increase immediately after only training session 1 (44 ± 52%). But 24 h after training session 1, post training session 2, 24 and 48 h after training 2, IL-10 concentrations were not statistically significant different (*P* > 0.05) from pre intervention. Interestingly, we evaluated the balance between Th2 and Th1 response with IL-10/IL-6 ratio, and this parameter showed a decrease of ~50%, 24 and 48 h after the session 2 when compared to baseline demonstrating a disruption in the balance of pro and anti-inflammatory cytokines following extreme conditioning program training.

With regard to OPG, it is a cytokine member of the TNF receptor superfamily that binds to two ligands, the receptor activator of nuclear factor kappa-B ligand (RANKL), a key cytokine for the differentiation of osteoclasts, and a ligand related to the induction of apoptosis (TRAIL), involved in immune surveillance. Although clinical results confirm that OPG is an active cytokine in a wide range of diseases (osteoporosis, arthritis, vascular calcification, bone cancer related disease; Sasso et al., 2015), there are still conflicting results regarding OPG and exercise. For example, Pereira et al. (2013) showed that an acute resistance training session (3 sets of 10 repetitions in whole body exercises with 60% of 1RM) induced no additional increase in pro-inflammatory cytokines nor a decrease in anti-inflammatory cytokines and OPG (measured before, immediately post and 60 min after exercise) in women with or without Metabolic Syndrome. On the other hand, Mezil et al. (2015) found that high intensity exercise (12 min on a cycle ergometer) of six 1-min intervals separated by 1-min rest intervals stimulates a rapid increase in pro-inflammatory cytokines (IL-1a: 40,3%; IL-1b: 41,3%; IL-6: 70%; and TNF-a: 76%) followed by a decline 24 h later, resulting in pro-inflammatory cytokine levels below baseline value. No significant exercise-induced changes in IL-10 were observed. Nevertheless, at 5 min post-exercise, OPG increased from baseline (13.5%), at 1 and 24 h post-exercise the values decreased. Furthermore, they observed a positive correlation between the percent decline in OPG and IL-10 at 24 h post-exercise in comparison to their values at 5 min and 1 h.

However, the main difficulties in comparing our results with previously published studies are that most studies were limited regarding the time-course analyses (1–24 h post exercise) of OPG and different exercise (resistance training vs. high intensity interval training) protocols were used. Nevertheless, individuals involved with any high intensity functional movement training program should be aware of these exacerbated cytokine responses to avoid functional overreaching, regardless of muscle power recovery.

Some limitations of the present study should be highlighted, such as the reduced number of individuals, lack of diet control and the absence of female participants and upper limb power measures. Finally, conducting a session with only 24 h of recovery may have been influenced the response of cytokines on subsequent WOD. Therefore, further studies comparing different intensities and WOD with adequate recovery days are needed to elucidate these answers. Nonetheless, future studies including muscle biopsies could improve understanding of the muscle environment and not only select systems such as the immune or metabolic systems.

## Conclusions

In conclusion, 2 consecutive days of extreme conditioning program training elicited a significant decrease in anti-inflammatory cytokines without impairments in muscle power. Further training and longitudinal investigations are necessary to determine the consequences of this finding. While we observed no negative effect on muscular power, it is still recommended that caution be exercised due to the suppressive effect 2 consecutive days of extreme conditioning program training had on the immune system. While future research is needed to determine the significance of this result, it is recommended that the incorporation of lower intensity sessions and/or resting days would help to minimize immune disturbances. This could be a particularly useful training strategy for individuals who are in an immunocompromised status (such as a chronic stress state, or returning from an acute bout of illness), or during specific parts of the year in which viruses tend to weaken the immune system.

## Author contributions

Conceived and designed the experiments: RT, DN, and JP. Performed the experiments: RT, DN, DV, JD, LD, IN, and JP. Analyzed the data: RT, NF, VD, ML, and ON. Contributed reagents/materials/analysis tools: RT, VD, ON, and ML. Wrote the paper: RT, NF, JN, DN, and JP.

### Conflict of interest statement

The authors declare that the research was conducted in the absence of any commercial or financial relationships that could be construed as a potential conflict of interest.
