# Corrigendum: Two Consecutive Days of Extreme Conditioning Program Training Affects Pro and Anti-inflammatory Cytokines and Osteoprotegerin without Impairments in Muscle Power

**DOI:** 10.3389/fphys.2018.00771

**Published:** 2018-07-16

**Authors:** Ramires A. Tibana, Leonardo M. de Almeida, Nuno M. Frade de Sousa, Dahan da Cunha Nascimento, Ivo V. de Sousa Neto, Jeeser A. de Almeida, Vinicius C. de Souza, Maria de Fátima T. P. L. Lopes, Otávio de Tolêdo Nobrega, Denis C. L. Vieira, James W. Navalta, Jonato Prestes

**Affiliations:** ^1^Graduation Program on Physical Education, Catholic University of Brasilia, Brasília, Brazil; ^2^Laboratory of Exercise Physiology, Faculty Estacio of Vitoria, Vitória, Brazil; ^3^Medical Sciences, Faculty of Medicine, University of Brasilia, Brasília, Brazil; ^4^UDF-Centro Universitário, Brasília, Brazil; ^5^Department of Kinesiology and Nutrition Sciences, University of Nevada, Las Vegas, NV, United States

**Keywords:** inflammatory response, weight training, extreme condition, muscle power, Overtraining

The final sentence in the legend of Figure 3 has been reworded to explicitly state the significance of the symbols. ^*^p < 0.05 comparing to Pre T1; ^†^*p* < 0.05 comparing to Post T1; ^‡^*p* < 0.05 comparing to 24 h Post T1. This clarification does not change the scientific conclusions of the article in any way.

The original version of the article has been updated following the highlighting of issues by a third party.

## Conflict of interest statement

The authors declare that the research was conducted in the absence of any commercial or financial relationships that could be construed as a potential conflict of interest.

